# Phase Formation and Thermal Stability of Reactively Sputtered YTaO_4_–ZrO_2_ Coatings

**DOI:** 10.3390/ma14030692

**Published:** 2021-02-02

**Authors:** Bastian Stelzer, Katrin Pingen, Marcus Hans, Damian M. Holzapfel, Silvia Richter, Joachim Mayer, Konda Gokuldoss Pradeep, Jochen M. Schneider

**Affiliations:** 1 Materials Chemistry, RWTH Aachen University, 52074 Aachen, Germany; katrin.pingen@rwth-aachen.de (K.P.); hans@mch.rwth-aachen.de (M.H.); holzapfel@mch.rwth-aachen.de (D.M.H.); schneider@mch.rwth-aachen.de (J.M.S.); 2 Central Facility for Electron Microscopy, RWTH Aachen University, 52074 Aachen, Germany; richter@gfe.rwth-aachen.de (S.R.); mayer@gfe.rwth-aachen.de (J.M.); 3 Department of Metallurgical and Materials Engineering, Indian Institute of Technology Madras, Chennai 600036, India; kgprad@iitm.ac.in

**Keywords:** yttrium tantalate, zirconia, thermal barrier coatings, PVD, phase stability

## Abstract

Y_(1−x)/2_Ta_(1−x)/2_Zr_x_O_2_ coatings with 0 to 44 mol% ZrO_2_ were synthesized by sputtering. Phase-pure *M’*-YTaO_4_ coatings were obtained at a substrate temperature of 900 °C. Alloying with ZrO_2_ resulted in the growth of *M’* along with *t*-Zr(Y,Ta)O_2_ for ≤15 mol%, while for ≥28 mol%, ZrO_2_ X-ray diffraction (XRD) phase-pure metastable *t* was formed, which may be caused by small grain sizes and/or kinetic limitations. The former phase region transformed into *M’* and *M* and the latter to an *M’ + t* and *M + t* phase region upon annealing to 1300 and 1650 °C, respectively. In addition to *M* and *t*, *T*-YTa(Zr)O_4_ phase fractions were observed at room temperature for ZrO_2_ contents ≥28 mol% after annealing to 1650 °C. *T* phase fractions increased during in situ heating XRD at 80 °C. At 1650 °C, a reaction with the *α*-Al_2_O_3_ substrate resulted in the formation of AlTaO_4_ and an Al-Ta-Y-O compound.

## 1. Introduction

Thermal barrier coatings (TBCs) are commonly used in gas turbines to protect metallic components from excessive temperatures. TBCs enable higher operating temperatures and thereby enhance performance and energy efficiency while improving durability [1,2]. ZrO_2_ alloyed with ≈8 mol% YO_1.5_ (YSZ) to stabilize the tetragonal structure is the most commonly used TBC nowadays [3,4]. To further improve the sustainability and lifetimes of turbine engine components, new materials for TBCs have to be identified, surpassing the high fracture toughness of approximately 40 J·m^−2^ and thermal stability of up to 1300 °C of YSZ while reducing thermal conductivity of approximately 2 W·m^−1^ K^−1^ as well as minimizing corrosion by molten deposits [2,4,5].

**Figure 1:**
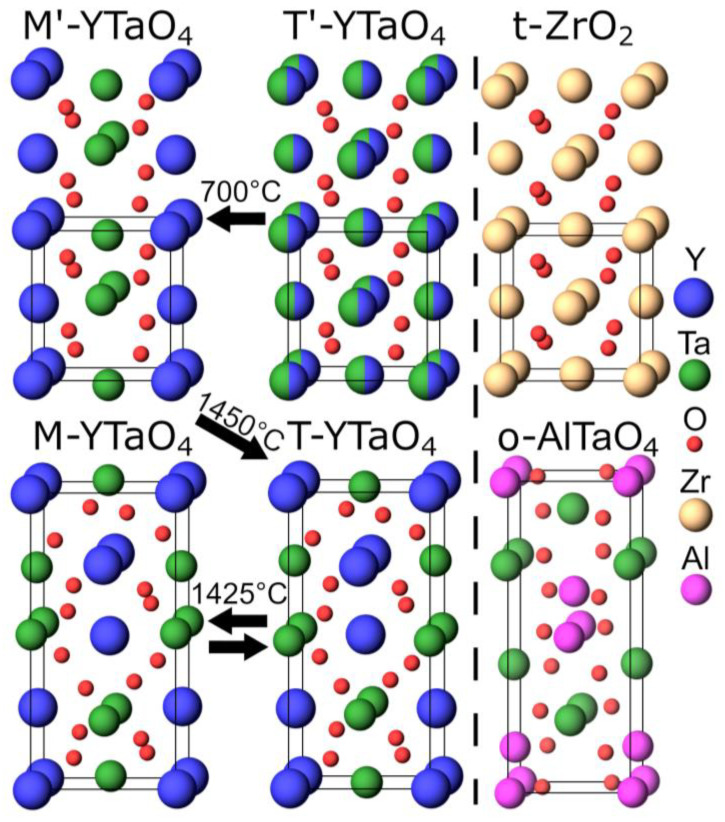
Structures of M′-, M-, T′-, T-YTaO_4_, t-ZrO_2_, and o-AlTaO_4_ phases with indicated transition pathways and temperatures based on experimental findings by [8,12,15].

The Y_2_O_3_–Ta_2_O_5_–ZrO_2_ system has gained attention as a promising candidate for improved TBCs. The pseudo binary YTaO_4_–ZrO_2_ is of particular interest due to reports of high fracture toughness [4,6] paired with lower thermal conductivity [5,7], superior thermal stability [4,8], and improved resistance to corrosion [4,9] compared to YSZ. YTaO_4_ is a polymorphous material reported to exhibit two different monoclinic [10,11] as well as two tetragonal structures [12]. The crystal structures of the phases are depicted in Figure 1. Heinze et al. [13] and Zhang et al. [14] predicted by ab initio approaches the monoclinic *M’* phase (space group P2/a) to be the thermodynamically stable configuration at 0 K. At approximately 1450 °C, a reconstructive phase transformation to a tetragonal scheelite structure *T* (space group I4_1_/a) occurs, altering the Ta coordination from six-fold to eight-fold [12,15]. The compound melts congruently at ≈2044 °C [16]. Upon cooling, the scheelite phase experiences a second-order displacive phase transition at 1426 ± 7 °C to the monoclinic *M* phase (space group I2) [6,8,10]. Hence, the *M* and *T* phases are structurally closely related. Phase formation modeling by Zhang et al. [14] revealed only a small difference in total Gibbs energies for *M’* and *M* of −0.121 kJ·mol^−1^, whereas a large energy barrier for the diffusion of Y of 3.26 eV is hindering the *M* to *M’* phase transition, indicating that *M* is a metastable phase, as the transformation to the thermodynamically stable *M’* phase appears to be kinetically limited. Mather and Davies [12] have observed the formation of a metastable *T’* phase (space group P4_2_/nmc) in between 700 and 850 °C during heating of initially amorphous samples. This phase was described as a tetragonal distorted form of cubic fluorite with random cation distribution [12]. However, this was not reproduced by others [15,17]. Whereas the *M* phase is considered as a potential TBC due to its low thermal conductivity [5,7,18] and ferroelastic toughening [4,6,8], *M’* is of interest as X‑ray phosphor in medical diagnostics because of its shorter Ta–O bonds, which enhance the charge-transfer processes utilized in luminescence [19,20].

**Figure 2:**
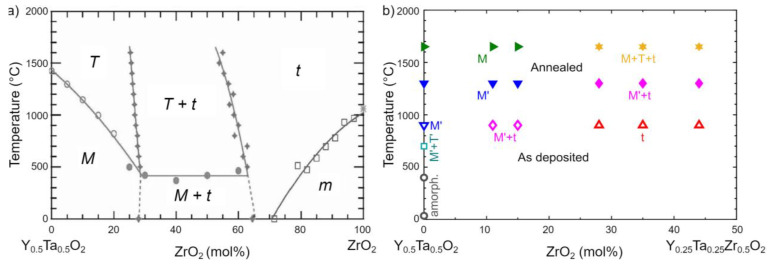
(**a**) Phase diagram along the YTaO_4_–ZrO_2_ pseudo binary adapted from Gurak et al. [21]; (**b**) Phases observed in this work for as-deposited samples (open symbols) synthesized at indicated substrate temperatures as well as samples annealed (filled symbols) at 1300 and 1650 °C for various ZrO_2_ mol%.

The influence of alloying ZrO_2_ to YTaO_4_ has previously been studied for sintered samples. Figure 2a depicts the pseudo-binary phase diagram of YTaO_4_–ZrO2 by Gurak et al. [21]. Along the pseudo-binary YTaO_4_−ZrO_2_, up to 25–28 mol% ZrO_2_ can be solved in *M*- as well as in *M’*-YTaO_4_ [15,21]. Gurak et al. reported that increasing ZrO_2_ concentrations lower the *M* to *T* transformation temperature to 450 ± 20 °C [21]. However, Flamant et al. observed the transformation temperature for the *M’* to *T* transformation to remain at approximately 1450 °C for varying ZrO_2_ contents [15]. On the Zr-rich side, *t*-Zr(Y,Ta)O_2_ (space group P4_2_/nmc) remains phase pure due to a composition of approximately 65 mol% ZrO_2_ [4,22]. Minor YTaO_4_ concentrations lead to the formation of monoclinic ZrO_2_ upon cooling [21,22]. Y^3+^ and Ta^5+^ stabilize the *t* phase, resulting in a non-transformable *t* phase region. As Zr^4+^ is substituted in equal parts by Y^3+^ and Ta^5+^, the net charge of the cations is compensated, and thus, other than for YSZ, no vacancy formation is necessary [23]. Hence, secondary phases are formed upon the excess of a few atomic percent of Y or Ta along the whole YTaO_4_–ZrO_2_ pseudo binary [24,25]. In between the *M* and *t* solid solutions exists a two-phase region of *M* and *t* up to 450 ± 20 °C with *M* transforming to *T* at higher temperatures [21].

There has been significant progress in characterizing the Y_2_O_3_–Ta_2_O_5_–ZrO_2_ system. However, studies are almost exclusively performed on sintered bulk samples or powders [4,5,7,8,15,16,17,18,19,21,22,24,25,26]. Physical vapor deposition (PVD) or thermal spraying are commonly employed for depositions of TBCs. Phase formation in PVD may vary significantly from observations made during typical bulk sintering techniques due to the enhancement of surface diffusion by impinging atoms and ions. Nevertheless, experimental work on electron beam (EB)–PVD coatings has only been published for Y_0.2_Ta_0.2_Zr_0.6_O_2_ [27] and other ZrO_2_-rich systems with partial [27] or complete [28] substitution of Y by Yb. In this work, the phase formation of sputtered Y_(1−x)/2_Ta_(1−x)/2_Zr_x_O_2_ coatings with ZrO_2_ contents from 0 to 44 mol% is studied systematically for the first time. To this end, a combinatorial reactive magnetron sputtering approach was employed to efficiently screen the effect of ZrO_2_ incorporation and substrate temperature on the phase formation as well as on the thermal stability.

## 2. Materials and Methods

**Figure 3:**
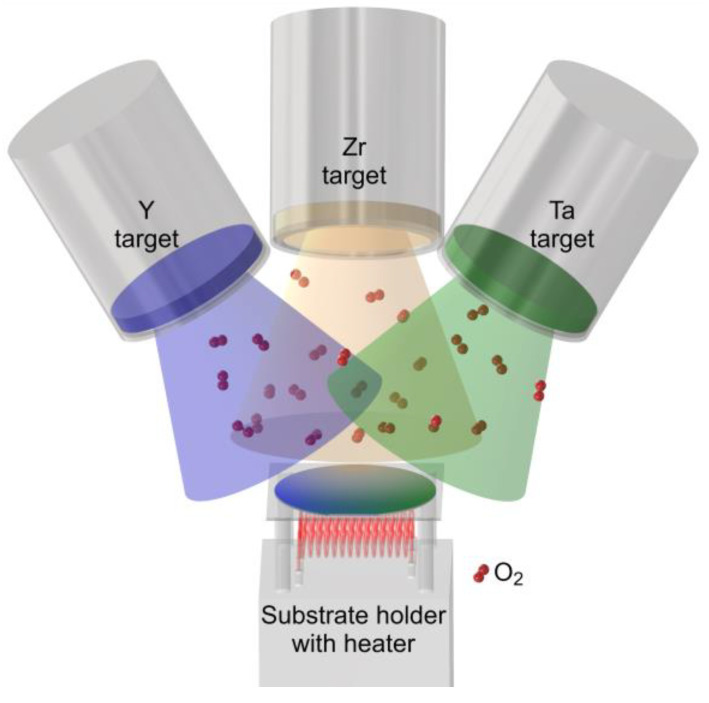
Schematic representation of deposition setup for reactive combinatorial magnetron sputtering of Y, Ta, and Zr.

Y_(1−x)/2_Ta_(1−x)/2_Zr_x_O_2_ coatings were synthesized by reactive direct current magnetron sputtering (DCMS) in a laboratory-scale deposition system by a combinatorial approach, as schematically depicted in Figure 3. 99.9% pure metallic Y, Ta, and Zr targets (50 mm diameter, 5 mm height) were located at a target to substrate distance of 100 mm. The single crystalline *α*-Al_2_O_3_ (0001) substrates (Siegert Wafer, Aachen, Germany) exhibited a diameter of 50.8 mm and were kept at floating potential. The target power density was set to 10.2 W cm^−2^ for Y and Ta, whereas it was varied from 0 to 10.2 W·cm^−2^ for Zr. The power was applied for a deposition time of 4 h using two ENI RPG-50E (MKS Instruments, Andover, MA, USA) and a MDX-10K (Advanced Energy, Fort Collins, CO, USA) power supplies. Samples were deposited without intentional substrate heating as well as at temperatures of 400, 700, and 900 °C. The base pressure at the employed substrate temperatures was below 2 × 10^−4^ Pa for all depositions. In order to obtain stoichiometric oxide films, the Ar (purity 6.0) and O_2_ (5.0) partial pressures were set to 0.1 and 0.3 Pa, respectively.

After deposition, approximately 4 × 4 mm² pieces were cut out of the combinatorial sample at a central location of the wafer with stoichiometric Y_(1−x)/2_Ta_(1−x)/2_Zr_x_O_2_ compositions. These samples were annealed in Ar (6.0) atmosphere in a Jupiter STA 449 C calorimeter (Netzsch, Selb, Germany). Heat treatments were performed for 1 h at a heating and cooling rate of 40 K·min^−1^. The temperatures of 1300 and 1650 °C were selected above the synthesis temperature of 900 °C, with one being below and one above M’/M to the T phase transition temperature.

The coatings were characterized regarding phase formation by X-ray diffraction (XRD) in an AXS D8 Discover (Bruker, Billerica, MA, USA) equipped with a General Area Detector Diffraction System (GADDS) using an incident angle of 15°. A Cu K_α_ radiation source was operated at a current and voltage of 40 mA and 40 kV, respectively. The reference XRD patterns employed for phase identification are given in Table 1. For in situ heating XRD experiments, an Anton Paar DHS 1100 heating stage with a graphite dome was installed into the diffractometer. In situ heating experiments were conducted in vacuum at a pressure below 10^−2^ mbar up to a sample temperature of 825 °C. A thermocouple was pressed onto the sample surface in order to measure sample temperatures. 

The chemical composition of the coatings was determined by energy-dispersive X-ray analysis (EDX) in a TM4000Plus scanning electron microscope (SEM) (Hitachi, Chiyoda, Japan) equipped with a Quantax75 EDX detector (Bruker, Billerica, MA, USA). For quantification, a reference measurement was performed by wavelength-dispersive X-ray spectroscopy (WDX) in a JXA-8530F microprobe (JEOL, Akishima, Japan) equipped with a field emission electron gun. Prior to the WDX measurement, a carbon coating was applied onto the samples to reduce electrostatic charging effects. An additional measurement on a non-carbon-coated part of the sample was carried out determining an upper limit for C contamination of ≈2.5 at.%. In this work, compositions are given as mole percent of the single cation formula units Y_0.5_Ta_0.5_O_2_ and ZrO_2_.

Spatially resolved chemical compositions were measured by laser-assisted three-dimensional atom probe tomography (3D-APT) in a local electrode atom probe 4000X HR (CAMECA, Madison, WI, USA). The YTaO_4_ thin film (0 mol% ZrO_2_) was measured with 50 pJ laser pulse energy, 125 kHz laser pulse frequency, and 60 K base temperature. Since these conditions resulted in immediate fracture of the thin films with 35 mol% ZrO_2_, the laser pulse energy and base temperature were reduced to 10 pJ and 30 K, respectively, for the Zr-containing thin films. The detection rate was set to 0.5% for all three measurements, and at least 5 million ions were acquired. Data analysis was carried out using IVAS 3.8.0.

Needle-like APT specimens were prepared in a standard lift-out procedure [29] by focused ion beam (FIB) in a Helios Nanolab 660 dual-beam microscope (FEI, Hillsboro, OR, USA). Furthermore, this dual-beam microscope was used for top-view imaging employing a back-scattered electron (BSE) detector as well as for preparation of lamellae and subsequent scanning transmission electron microscopy (STEM) using a STEM III detector. Chemical compositions of lamellae were measured by standardless EDX with an Octane Elect Plus (EDAX, Mahwah, NJ, USA).

The arithmetic mean roughness was acquired in a VK-9700 laser optical microscope (Keyence, Osaka, Japan). Nanoindentation measurements were performed on a TI-900 TriboIndenter (Hysitron, Minneapolis, MN, USA) equipped with a Berkovich diamond tip (Hysitron, Minneapolis, MN, USA). The tip area function was calibrated on fused silica. The applied load of 1200 µN resulted in maximum indentation depths below 10% of the film thickness. The elastic modulus was determined according to the method by Oliver and Pharr [30] for a minimum of 49 indents per sample. A Poisson’s ratio of 0.29 [14] was used.

## 3. Results and Discussion

### 3.1. Effect of Substrate Temperature on YTaO_4_ Depositions

**Figure 4:**
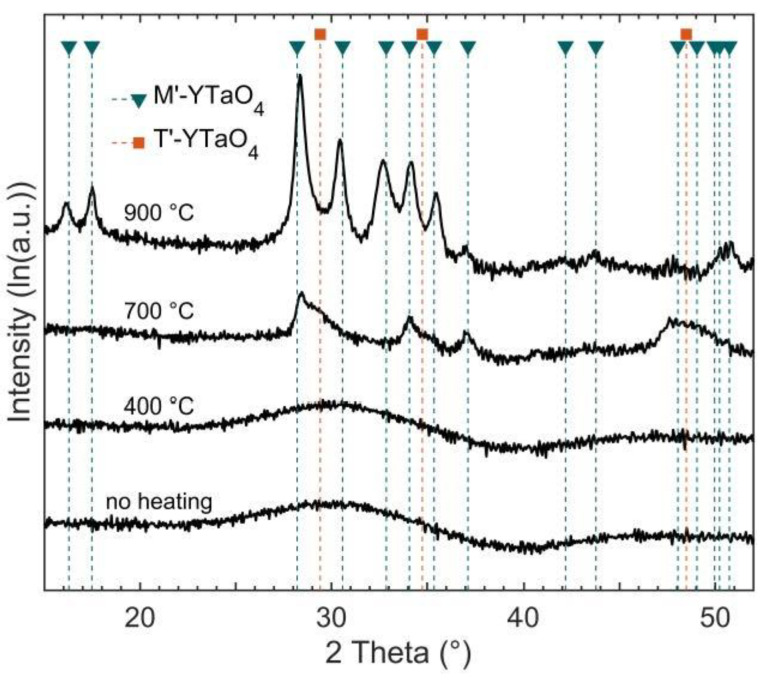
Diffractograms of YTaO_4_ coatings deposited at indicated substrate temperatures.

The phase formation of the as-deposited YTaO_4_ coatings without intentional heating as well as at substrate temperatures of 400, 700, and 900 °C was investigated by XRD as depicted in Figure 4. For the diffraction experiments, the as-deposited combinatorial coatings were screened for locations exhibiting Y to Ta ratios of 1 based on EDX measurements employing the WDX reference measurement as standard. 

Coatings deposited at substrate temperatures of 400 °C or below are XRD amorphous. At a substrate temperature of 700 °C, several broad peaks consistent with the reported peak positions of *M’* appear. The *M’* (−111) and (200) peaks at 28.4 and 34.0° exhibit broadening toward larger 2*θ* values, suggesting the presence of the metastable *T’*-phase. At a nominal substrate temperature of 900 °C, crystallinity is further improved with all peaks coinciding with peak positions of *M’*. Due to a preferred (−111) orientation of the sample, several peaks such as the (011) and (110) are only observed after tilting the sample (not shown here). Hence, the results indicate the formation of an XRD phase-pure M’ coating. These observations are in agreement with Mather and Davies [12] who identified an XRD amorphous structure at 600 °C, *T’* formation at 800 °C, and a phase mixture of *T’* and *M’* at 900 °C for sol–gel-prepared YTaO_4_. In this work, the phase mixture of *M’* and *T’* was already observed at 700 °C. It is reasonable to assume that ion bombardment-induced surface diffusion occurring during sputtering causes the reduced phase formation temperature compared to bulk diffusion dominated processes commonly employed in previous studies. This has previously been observed for Mo_2_BC [31] as well as Cr_2_AlC [32,33]. Furthermore, the formation of an XRD phase-pure *M’* coating is observed at 900 °C, which is consistent with the literature, as the formation of *M’* instead of *M* is widely reported for synthesis methods below the *M’–T* transformation temperature of ≈1450 °C [10,12,15].

**Figure 5:**
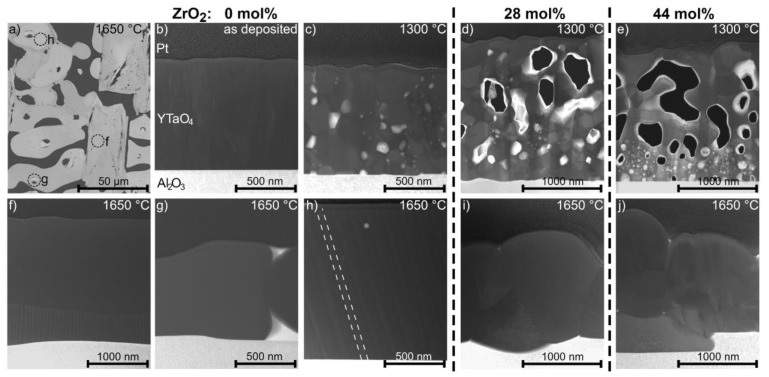
(**a**) Top-view SEM image of ZrO_2_ free sample after annealing at 1650 °C; Dark field STEM images of cross-sections prepared by focused ion beam (FIB) milling of as-deposited (**b**) as well as annealed for 1 h at 1300 °C (**c**–**e**) and 1650 °C (**f**–**j**) for 0, 28, and 44 mol% ZrO_2_. Locations of cross-sections for (**f**–**h**) are indicated in a. Dashed lines in h highlight a twin.

**Figure 6:**
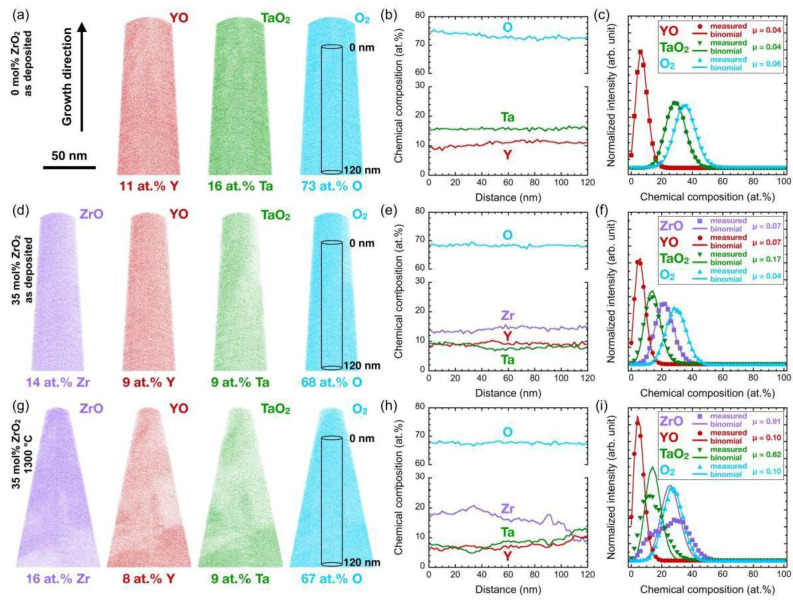
Spatially resolved chemical composition analysis by atom probe tomography (APT) of as-deposited samples with 0 mol% ZrO_2_ (**a**–**c**) and 35 mol% ZrO_2_ (**d**–**f**) as well as a sample with 35 mol% ZrO_2_ annealed at 1300 °C (**g**–**i**); Reconstructions of molecular ions in (**a**,**d**,**g**) are provided as 20 nm slices with a length of 150 nm; Concentration profiles within cylinders indicated in (**a**,**d**,**g**) are shown in (**b**,**e**,**h**); Frequency distribution analyses with Pearson correlation coefficients µ are given in (**c**,**f**,**i**), and the measured distributions (data points) are compared to a random, binomial distribution (lines).

The coating deposited at 900 °C exhibits a thickness of 970 ± 5 nm with a dense, fibrous microstructure, as shown in the STEM image in Figure 5b. APT measurements (Figure 6a–c) indicate a random distribution of Y, Ta, and O with Pearson correlation coefficients *µ* ≤ 0.06 within the analyzed 1.9 × 10^6^ atoms [34]. Hence, APT analysis supports the notion of the exclusive formation of the YTaO_4_ phases (*T’*, *M’*, *M*, or *T*) without precipitates of Y_3_TaO_7_ or YTa_7_O_19_, which have been reported to form for Ta or Y deficiencies of few atomic percent [24,26].

### 3.2. Effect of Zirconia Alloying on Phase Formation of As-Deposited Coatings

**Figure 7:**
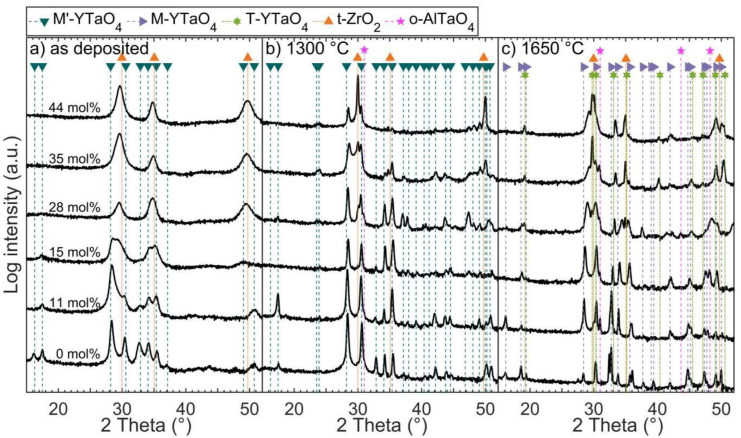
Diffractograms of Y_(1−x)/2_Ta_(1−x)/2_Zr_x_O_2_ coatings with x ranging from 0 to 44 in (**a**) As-deposited, (**b**) Annealed for 1 h at 1300 °C, and (**c**) Annealed for 1 h at 1650 °C states.

Y_(1−x)/2_Ta_(1−x)/2_Zr_x_O_2_ coatings were deposited at 900 °C with varying Zr content and were subsequently analyzed by XRD at EDX measured chemical compositions of 0, 11, 15, 28, 35, and 44 mol% ZrO_2_, as shown in Figure 7a. The thickness for the samples deposited at a constant deposition time of 4 h range from 970 ± 5 nm (4.0 nm/min) for the coating without Zr up to 1423 ± 4 nm (5.9 nm/min) for the sample with 44 mol% ZrO_2_, with the deposition rate given in brackets. The low deposition rates are expected to be caused by the targets running in poisoned mode and the possible thermal evaporation from the substrate due to the high temperature. 

As described above, coatings deposited at 900 °C with 0 mol% ZrO_2_ exhibit XRD phase-pure *M’*-YTaO_4_. For increasing ZrO_2_ contents up to 15 mol%, the formation of an *M’* solid solution phase with declining diffracted intensities and a peak shift of the (−111) peak at 28.4° toward larger diffraction angles is observable. Zr was reported to equally substitute Y and Ta in the monoclinic YTaO_4_ structures, where the slightly smaller ion radius of Zr^4+^ (8.4 Å) compared to the average ion radius of Y^3+^ (10.2 Å) and Ta^5+^ (7.4 Å) [35] being 8.7 Å results in a reduction of the unit cell volume and thus a peak shift toward larger diffraction angles. The presence of a tetragonal second phase is apparent at 15 mol% ZrO_2_ and may already be formed for 11 mol% ZrO_2_, as indicated by the pronounced shoulder of the (−111) peak toward larger 2*θ* values. Hence, the solubility limit of ZrO_2_ in as-deposited *M’* solid solutions appears to be <15 mol%, which is significantly lower compared to the solubility limit of 25 to 28 mol% ZrO_2_ reported for sintered *M’* samples by Flamant et al. [15].

Further increase of ZrO_2_ to 28 mol% and up to the maximum synthesized concentration of 44 mol% ZrO_2_ results in the formation of a phase-pure *t*-Zr(Y,Ta)O_2_ solid solution. The formation of a two-phase region of *t* in combination with *M* or *M’* phase has been reported for sintered samples with ≈25 to ≈65 mol% ZrO_2_ [10,12,15,16,17,18]. However, Van Sluytman et al. [27] deposited tetragonal Y_0.2_Ta_0.2_Zr_0.6_O_2_ coatings by EB-PVD consisting of a *t*-phase matrix with *T*-phase precipitates with distinguishable chemical compositions. APT analysis of the thin film with 35 mol% ZrO_2_ (Figure 6d–f) showed no major segregation of Y, Ta, or Zr in the analyzed 1.5 × 10^6^ atoms, supporting the notion of a single-phase coating. Coatings deposited in this work exhibit phase-pure *t* in the YTaO_4_–ZrO_2_ system to an unprecedented low ZrO_2_ content of 28 mol%.

Additions of Y and Ta into ZrO_2_ are well known to stabilize the high-temperature tetragonal phase *t*-ZrO_2_ and suppress monoclinic *m*-ZrO_2_, as depicted in the phase diagram in Figure 2a [4,21,22,36]. Kim et al. reported tetragonal Y_(1−x)/2_Ta_(1−x)/2_Zr_x_O_2_ to be stable independent of grain size for 78 to 84 mol% ZrO_2_, with analyzed grain sizes of up to 5 µm [22]. This formation of the *t* phase was explained by the introduction of local distortions due to the substitution of Zr by smaller Ta^5+^ and larger Y^3+^ cations [4] as well as low grain sizes, as was shown by Shukla and Seal [23]. *T’* was reported for the Y and Ta rich side of the YTaO_4_–ZrO_2_ pseudo-binary by Mather and Davies [12], who observed Zr free *T’*–YTaO_4_ at temperatures of approximately 800 °C. They described *T’* as a metastable tetragonal phase isostructural to *t* but with disordered cation distribution, see Figure 1, and speculated that this phase is stabilized by the Gibbs–Thomson effect. Small grain sizes are known to reverse the phase formation behavior during thin film synthesis compared to bulk processing, resulting in the stabilization of e.g., *γ*^−^ over *α*-Al_2_O_3_ [37], wurtzite over face-centered cubic (Ti, AlN) [38] as well as *t* over *m* in unalloyed ZrO_2_ [23].

In our work, the formation of both *m*-ZrO_2_ as well as *M’-YTaO_4_* is hindered. Whereas the suppression of *m* may be due to alloying with Y and Ta, it can be speculated that the suppression of *M’* is caused by small grain sizes, as was suggested by Mather and Davies [12] and/or by kinetically limited phase formation during magnetron sputtering. Evaluation of the broad diffraction peaks by Scherrer equation [39] yields crystallite sizes in between 15 ± 3 and 21 ± 6 nm for as-deposited phase-pure *t*‑Zr(Y,Ta)O_2_ solid solutions. Furthermore, the employed synthesis temperature of 900 °C and the minute ion bombardment during coating deposition at floating potential is expected to result in kinetically limited growth, causing the formation of the metastable phases. However, while this is in line with experiences from other material systems [40] and with the observations made in this work, the data at hand does not provide irrevocable evidence for one of the mechanisms mentioned above to dominate the others.

### 3.3. Effect of Annealing on Zirconia Alloyed YTaO_4_ Coatings

Annealing for 1 h at 1300 and 1650 °C resulted in significant changes in ex situ measured diffractograms for all the compositions studied here (Figure 7b). For the sample without ZrO_2_, the diffractogram obtained after annealing at 1300 °C shows an increase in crystallinity reflected in a decrease in full width at half maximum, while all peaks indicate the presence of *M’*, as it is the case for as deposited coatings. However, the texture of the film has changed, e.g., the (010) peak at 16.2° is not visible. Cross-sectional STEM imaging of the as-deposited and 1300 °C annealed sample is shown in Figure 5b,c. The microstructure changes from fibrous grains growing perpendicular to the surface in the as-deposited state to a morphology containing larger, globular grains and a significant amount of pores that evolve at the grain boundaries. Annealing at 1650 °C resulted in further grain growth, with single grains expanding over the full height of the coating (Figure 5h). Furthermore, the observed grains exhibit twin domains after annealing at 1650 °C. These grains cover several µm of the substrate as visible in top-view SEM imaging in Figure 5a. Additionally, SEM and STEM revealed local dewetting of the sapphire substrate after heat treatment at 1650 °C. The dark regions in Figure 5a stem from the *α*-Al_2_O_3_ substrate, which is exposed due to dewetting. Melting of any composition within the YO_1.5_–TaO_2.5_ system is not expected at 1650 °C according to phase diagrams proposed by Fernandez et al. [26] and Zhang et al. [14]. Hence, a solid-state dewetting process driven by differences in surface and interface energies and enabled by surface diffusion [41] is responsible for the exposure of the substrate. 

XRD analysis revealed the transformation from *M’* to *M* after annealing at 1650 °C (Figure 7c). The diffractogram of the ZrO_2_ free coating annealed at 1650 °C exhibits peaks at 27.5°, 32.4°, and 36.1°, not fitting to the *M*-phase. These may be linked to the observation of rectangular-shaped grains, shown in the SEM image in Figure 5a. EDX measured chemical compositions of a lamellae cut out of the arbitrary rounded shaped grains in Figure 5a are consistent with YTaO_4_. However, a lamella prepared from a rectangular-shaped grain contains a twinned grain with a composition fitting to YTaO_4_ above the *α*-Al_2_O_3_ substrate and on top, a grain containing Al in addition to Ta-Y-O (Figure 5f). These results suggest a reaction of the Y-Ta-O coating with the *α*-Al_2_O_3_ substrate. The actual phase is yet to be identified, and there are no reports on a structure corresponding to the observed Y-Ta-Al-O composition available. 

YTaO_4_ samples alloyed with 11 and 15 mol% ZrO_2_ and annealed at 1300 °C do not exhibit a peak broadening of the (−111) peak toward larger angles, as was seen for as deposited coatings. Thus, XRD indicates the formation of single-phase *M’* coatings after annealing. Likewise, heat treatment at 1650 °C results in the formation of *M*.

Annealing of as deposited XRD phase-pure *t* samples with ZrO_2_ contents of 28 mol% or higher at 1300 °C resulted in the formation of *M’* next to *t* (Figure 7c). An increase in ZrO_2_ leads to an increase in the *t* peak intensity at a 2*θ* value of ≈30.0°, which evolves from a shoulder of the (111) *M’* peak to the most prominent peak. The transformation from an as-deposited XRD single-phase *t* to a two-phase coating is in good agreement with the observation of Zr-rich as well as Y- and Ta-rich regions with reduced Zr content observed by APT for 35 mol% ZrO_2_ only after annealing. This segregation is mirrored by an increase in the Pearson correlation coefficients for ZrO from 0.07 to 0.91 in as-deposited and 1300 °C annealed state, respectively (Figure 6d–i). Correspondingly to previously discussed lower ZrO_2_ contents, heat treatment at 1650 °C resulted in the transformation of *M’* into *M*. However, the *t*-phase remains stable. Furthermore, a second tetragonal phase, the high temperature *T* phase, is observed after cooling.

**Figure 8:**
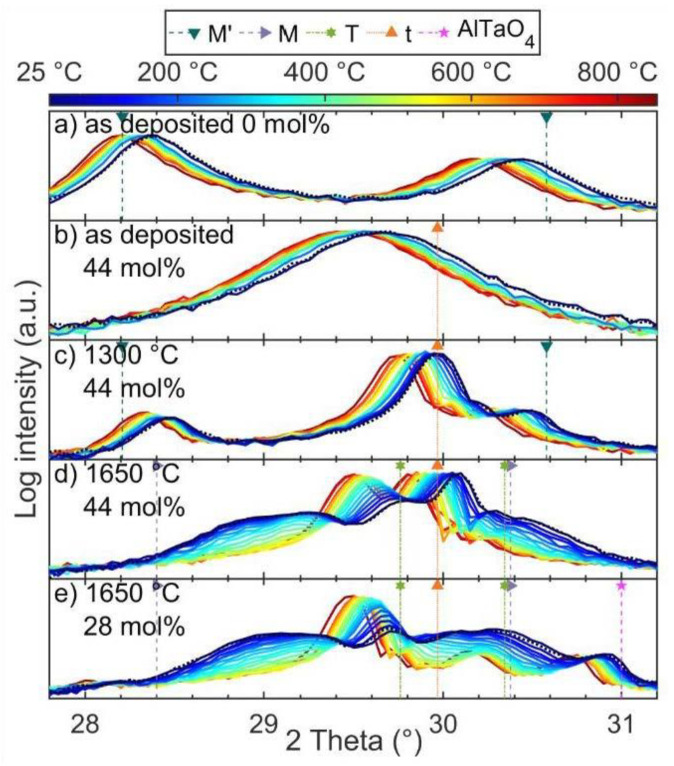
Diffractograms obtained during in situ heating experiments of as-deposited and pre-annealed Y_(1−x)/2_Ta_(1−x)/2_Zr_x_O_2_ coatings. Dotted intensities represent measurements at room temperature after completed in situ heating.

In situ heating XRD measurements up to approximately 825 °C were performed in order to study the thermal stability of both as-deposited and annealed coatings (Figure 8). For as deposited coatings with 0 and 44 mol% ZrO_2_ consisting of *M’* and *t*, respectively, in situ heating did not result in measurable changes of the constitution up to 825 °C but only in peak shifts due to thermal expansion. As no changes of the pre- and post-annealed coatings could be identified at room temperature, the obtained coatings are considered to be stable up to a temperature of 825 °C. The same observation was made for an *M’* and *t* two-phase coating with 44 mol% ZrO_2_ after annealing at 1300 °C (Figure 8a–c). Coatings pre-annealed at 1650 °C with ZrO_2_ contents of 28 and 44 mol% ZrO_2_ showed a more complex behavior. At room temperature, the 44 mol% ZrO_2_-containing sample, which was annealed at 1650 °C, exhibits a *T* as well as a *t* peak at 29.8° and 30.1°, respectively, and broader *M* peaks at 29.1° and 30.4° (Figure 8d). Upon heating, both *M* peaks exhibit a decrease in intensity already at temperatures of 80 °C, while the *T*-phase peak gains intensity. The intensity of the *t* peak is unaffected by the increase in temperature. At a temperature of approximately 480 °C, both *M*-phase peaks have disappeared, while the *T* peak ceases to gain intensity upon further heating. A second *T*-phase peak emerged at 30.1° out of the shoulder of the *t* and declining *M*-phase peak. Upon cooling, all observed phase transformations are fully reversible. For a ZrO_2_ concentration of 28 mol%, a similar decrease and increase of the *M* and *T* phase were observed (Figure 8e). The peak corresponding to *t*, barely identifiable at room temperature, is clearly visible after *M* transformed into *T* upon heating. 

Furthermore, a peak at 30.9° with temperature-independent intensity at room temperature was observed. For various compositions annealed at 1650 °C as well as for the coating composed of 28 mol% ZrO_2_ annealed at 1300 °C, this additional peak at ≈30.9° was obtained in XRD diffractograms (Figure 7b,c). This may be ascribed to orthorhombic AlTaO_4_. Grains with a Ta-Al-O composition have also been observed in lamellae cut out of the vicinity of an YTaO_4_ grain for the ZrO_2_ free sample annealed to 1650 °C (Figure 5g). A significant increase in roughness at the substrate–coating interface was observed for grains exhibiting a Ta-Al-O or the previously discussed Ta-Y-Al-O composition (Figure 5f,g,i,j). To a lower extent, this roughness enhancement is also observable after annealing to 1300 °C (Figure 5c–e). However, this is not observed for as-deposited coatings or areas covered with Al free *M* grains, as depicted in Figure 5h. Hence, it is inferred that the roughness enhancement is a consequence of the reaction of the coating with the *α*-Al_2_O_3_ substrate during annealing.

EDX measured compositions of both of the above described Al containing impurity phases indicate Y depletion. Thus, the formation of these phases may be caused by deviations from the Y_(1−x)/2_Ta_(1−x)/2_Zr_x_O_2_ composition. By their nature, combinatorially deposited coatings exhibit chemical gradients. Only comparably small 4 × 4 mm^2^ pieces were annealed in this study in order to limit diffusion effects and influences of heavily over- or understoichiometric regions. However, even this small sample size allows chemical gradients of approximately ±1 at.%. Hence, the reaction with *α*-Al_2_O_3_ may or may not be caused by combinatorial deposition-induced composition deviations from the YTaO_4_ stoichiometry. As TBCs are most commonly applied on thermally grown oxides consisting of *α*-Al_2_O_3_ [42], reactions between YTaO_4_ and *α*-Al_2_O_3_ may be critical for applications. Thus, the analysis of the obtained phases with emphasis on their high-temperature behavior should be addressed in future work.

Results of the thermal stability studies are summarized in Figure 2b. As-deposited coatings, including the supersaturated XRD single-phase *t* coatings, are stable up to at least 825 °C. Upon annealing to 1300 °C, *M’* remains stable, and for 28 mol% ZrO_2_ and above, a two-phase region with *M’* and *t* is observed. After annealing to 1650 °C, the *t* phase remains unaffected, while *M’* transforms to *M*. *M’* is reported to transform to the high-temperature *T* phase at approximately 1450 °C [15]. Upon cooling, the structurally similar *M* phase is formed. However, for coatings with 28 to 44 mol% ZrO_2_, *T* was partly retained down to room temperature. This behavior is independent of the cooling rate, 40 K/min in case of the 1650 °C annealing procedure compared to an average cooling rate of approximately 4 K/min for in situ heating XRD studies. Zr is known to promote the high-temperature tetragonal structure of YTaO_4_, as shown by reduced *M–T* transformation temperatures [21]. Previously, Gurak et al. [21], Shian et al. [8], as well as van Sluytman et al. [27] were able to retain fractions of *T* at room temperature for Zr containing YTaO_4_ after annealing. Along with the stabilization of *T* to room temperature, the *M* to *T* transformation temperature was significantly lowered. Reduced *M*-phase peak intensities along with increasing *T*-phase peak intensities were observable after initial heating to 80 °C, which is well below the previously reported temperature range of 250 °C to 450 ± 20 °C [21] for the same compositional range. On the other hand, the *M’* to *T* transformation temperature was observed to be in between 1300 and 1650 °C for all analyzed ZrO_2_ contents, which is in excellent agreement with observations by Flamant et al. [15] reporting the *M’* to *T* transformation temperature to be independent of ZrO_2_ at approximately 1450 °C. 

*M’* as well as *M* coatings without *t* solid solution were obtained for 0 to 15 mol% ZrO_2_ after annealing to 1300 and 1650 °C, respectively. Hence, similar solubility limits of ZrO_2_ in *M* and *M’* in between 15 and 28 mol% are derived. These are in good agreement with the solubility limits of *M’* and *M* to be in the range of 25–28 mol% ZrO_2_ found in literature [15,21]. However, due to the here observed Al containing impurity phases, an unambiguous statement on the solubility limit of *M* and *M’* is not feasible. 

**Figure 9:**
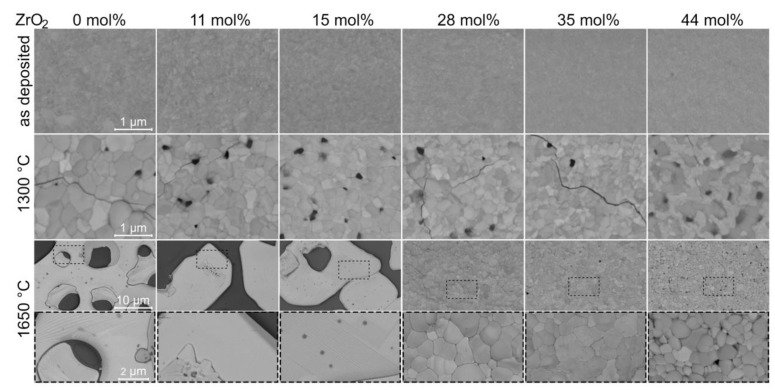
Top view of Y_(1−x)/2_Ta_(1−x)/2_Zr_x_O_2_ coatings by SEM with 0 to 44 mol% ZrO_2_ in as-deposited, annealed for 1 h at 1300 °C, and annealed for 1 h at 1650 °C conditions. For samples annealed at 1650 °C, two magnifications are shown.

Grain sizes of as-deposited as well as samples annealed at 1300 and 1650 °C are decreasing with increasing ZrO_2_ content, as can be seen in the top-view SEM images depicted in Figure 9. This is in line with observations on sintered samples by Shian et al. [8]. All single-phase *M* coatings annealed at 1650 °C exhibit grains covering several tenths of µm of the substrate, while leaving other substrate regions uncovered. Coatings annealed at 1650 °C with ZrO_2_ contents of 28 mol% or higher exhibit reduced grain sizes and no dewetting of the substrate. The formation of *t* as a second phase may inhibit grain growth. The correlations of grain sizes with ZrO_2_ contents are also observed in cross-sectional STEM imaging (Figure 5h–j). Furthermore, the large variation in grain sizes results in significant differences in roughness. The arithmetical mean roughness *R_a_* range for ZrO_2_ free samples is from 10 ± 2 nm to 739 ± 87 nm and for 44 mol% ZrO_2_, it is from 14 ± 3 nm to 172 ± 14 nm for the as-deposited and 1650 °C annealed state, respectively.

For all coatings annealed at 1650 °C, twinned grains were observed by top-view SEM (Figure 9) and cross-sectional STEM (Figure 5h,i). Twinning is a known characteristic of a ferroelastic response to stresses [43]. Hence, the here observed twins are expected to be induced by thermal stresses in ferroelastic *M* [6,8], resulting in (ferroelastic) toughening of *M*-containing samples. For increased ZrO_2_ concentrations, a reduction of the twin population is observed, which correlates with the observation of lower *M* and higher *T* phase fractions at room temperature. No twinning and hence no toughening is observed for samples annealed at 1300 °C. Consequently, cracks of various sizes evolved in all of these samples (Figure 9), although these *M’*-containing samples were exposed to a 350 °C lower annealing temperature and thus are expected to exhibit significantly smaller thermal strains compared to coatings exhibiting *M*. Crack formation may also be enabled by the presence of pores along grain boundaries as observed in cross-sectional STEM images for all analyzed samples annealed at 1300 °C (Figure 5c–e). Hence, the results indicate a superior behavior of the *M* phase as a TBC compared to *M’* coatings.

### 3.4. Elastic Properties of As-Deposited Coatings

**Figure 10:**
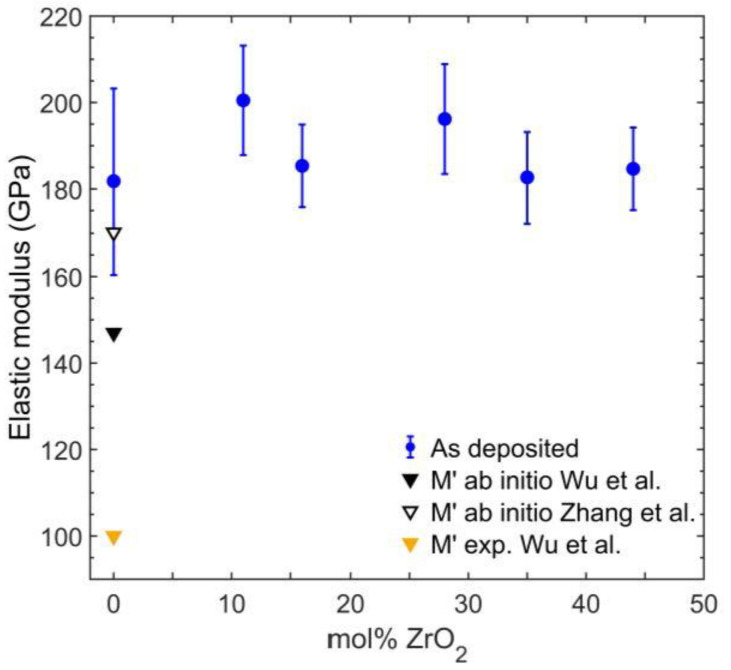
Elastic modulus measured by nanoindentation as a function of ZrO_2_ content for as-deposited coatings (blue). Black symbols indicate ab initio calculated elastic moduli by Wu et al. (filled symbol) [44] and Zhang et al. (open symbol) [14] for M’. Experimental results by Wu et al. [44] are shown in orange.

The elastic modulus was assessed by nanoindentation. Resulting elastic moduli in dependence of ZrO_2_ are depicted in Figure 10. As-deposited ZrO_2_ free *M’* coatings yielded an elastic modulus of 182 ± 21 GPa, with the error given as the standard deviation of all measured moduli. Reported results of ab initio calculations of the elastic modulus of *M’* vary considerably. While Wu et al. [44] obtained a value of 147 GPa, Zhang et al. [14] calculated the elastic modulus to be 170.2 GPa. Hence, the measured elastic modulus of the as-deposited coatings is significantly higher than the calculated value by Wu et al. but agrees within the error of the measurement with ab initio results by Zhang et al. Wu et al. also reported an experimentally derived elastic modulus of 100 GPa determined by nanoindentation for spark plasma sintered *M’* [44]. The higher elastic moduli in this work may be rationalized by a higher density of the samples and by compressive stresses common for PVD-deposited coatings [45]. Additions of ZrO_2_ did not show a significant impact on the measured elastic moduli. Due to the increase in roughness after annealing, no reliable nanoindentation measurements were feasible.

## 4. Conclusions

Y_(1−x)/2_Ta_(1−x)/2_Zr_x_O_2_ coatings with ZrO_2_ contents ranging from 0 to 44 mol% were synthesized by reactive DCMS. For ZrO_2_ free YTaO_4_, a substrate temperature of 900 °C resulted in the formation of XRD phase-pure *M’* coatings. The addition of 11 to 15 mol% ZrO_2_ led to the formation of tetragonal *t*-Zr(Y,Ta)O_2_ next to *M’*. XRD phase-pure *t* coatings were deposited for unprecedentedly low ZrO_2_ contents of 44 and down to 28 mol%. The formation of the metastable tetragonal structure may be mediated by small crystallite sizes in the range of 20 nm and/or kinetic limitations during growth.

Whereas *M’* remained stable after annealing at 1300 °C for 1 h, *M* was formed after annealing at 1650 °C. XRD phase analysis after annealing revealed solubility limits of ZrO_2_ in *M’* as well as *M* in-between 15 and 28 mol%, whereas as-deposited coatings including metastable phases exhibit a solubility limit of ZrO_2_ in *M’* < 15 mol%. As deposited phase-pure *t* samples obtained for ZrO_2_ ≥ 28 mol% transformed to *M’ + t* and *M + t* phase regions after annealing to 1300 and 1650 °C, respectively. Furthermore, for these compositions, *T* was observed at room temperature after annealing to 1650 °C. This *T* phase fraction increased in in situ heating XRD experiments at temperatures as low as 80 °C, with the transition of *M* phase into *T* completed at 480 °C.

Crack formation was observed by SEM imaging for all coatings after annealing at 1300 °C. It may be speculated that this is induced by the thermal expansion mismatches between the α-Al_2_O_3_ substrate and the M’ coatings. Coatings annealed at 1650 °C, containing *M*-phase, showed twinning but no crack formation, which may be rationalized by the ferroelastic behavior of *M*. For ≤ 15 mol% ZrO_2_, annealing to 1650 °C led to partial dewetting of the substrate. Furthermore, heat treatment at 1650 °C gave rise to a reaction of the deposited coating with the *α*-Al_2_O_3_ substrate independent of ZrO_2_ contents. This resulted in the formation of orthorhombic AlTaO_4_ and a yet unknown Ta-Y-Al-O compound. Hence, the here presented results motivate further analysis of the interaction of *M*-YTa(Zr)O_4_ solid solutions and *α*-Al_2_O_3_ to assess the suitability of YTaO_4_ as TBC on aluminides.

## Figures and Tables

**Table 1:** List of abbreviations for phases including compound, space group, and International Centre for Powder Diffraction (ICDD) Power Diffraction File (PDF) number or literature for used XRD references.

Abbreviation	Compound	Space Group	PDF Number
*M’*	YTaO_4_	P2/a	00-024-1425
*M*	YTaO_4_	I2	00-024-1415
*T’*	YTaO_4_	P4_2_/nmc	00-050-0846
*T*	YTaO_4_	I4_1_/a	Feng et al. [6]
*T*	ZrO_2_	P4_2_/nmc	00-043-0308
*O*	AlTaO_4_	Pbcn	01-079-2410

## Data Availability

The data presented in this study are available on request from the corresponding author.
